# An Observational Study of Anticipatory Coping Behavior of Women for Chemotherapy-Induced Alopecia for Breast Cancer Treatment

**DOI:** 10.7759/cureus.29097

**Published:** 2022-09-12

**Authors:** Deepak Raj G, Oseen Shaikh, Uday Kumbhar, Sudharsanan Sundaramurthi, Vikas Menon, Chellappa Vijayakumar

**Affiliations:** 1 Surgery, Jawaharlal Institute of Postgraduate Medical Education and Research, Puducherry, IND; 2 Psychiatry, Jawaharlal Institute of Postgraduate Medical Education and Research, Puducherry, IND

**Keywords:** chemotherapy-induced alopecia, alopecia, chemotherapy, breast cancer, anticipatory coping behavior

## Abstract

Background

It is necessary to explore patients' expectations of chemotherapy-induced alopecia, anticipate reactions to alopecia, and how women intend to prepare for an altered body appearance. Studies regarding women's critical aspects of anticipatory coping behavior (anticipate reactions to alopecia and how women intend to prepare for a modified body appearance) towards hair loss and factors influencing it are sparse, especially from India. This study helped identify the factors influencing the anticipatory coping behavior toward chemotherapy-induced alopecia.

Methodology

This was a cross-sectional analytic study carried out for three months, including all breast cancer patients admitted for neo-adjuvant chemotherapy. Anticipatory coping behavior before and during each chemotherapy cycle period (minimum one cycle) was assessed using the World Health Organization scale. Study patients received two sets of questionnaires. The first question is asked before the start of chemotherapy, and the second is requested at least four weeks after completing the first chemotherapy. The first question included baseline demographic data, disease details, and awareness about hair loss. The second question had hair loss pattern, severity of cancer-induced alopecia, and rated the degree of alopecia in a visual analog scale score. Individual psychosocial and social factors associated with the anticipatory coping behavior were documented using a self-administered questionnaire.

Results

A total of 40 patients were included in the study. All of the participants reported anticipating hair loss. Five percent of the patients came to terms with the inevitability of hair loss. Around 22.5% of them became ready, 67.5% of the patients took control and 52.5% of the patients felt shame in front of society due to a lack of anticipatory behavior. About 47.5% of the participants felt that not being willing to wear a wig is a reason for deficient anticipatory coping behavior.

Conclusion

The study patients' main reasons for lack of anticipatory coping behavior were shame in society, insecure thoughts about the future, and unwillingness to live with a wig. It should be advised to such patients that losing hair is not a health problem (though it is an emotional issue), and clinicians can encourage them to use hats. Further multi-institutional prospective studies are required to assess the poor motivation of health care professionals to achieve target anticipatory coping behavior.

## Introduction

Chemotherapy-induced alopecia (CIA) affects each individual differently. For some patients, the fear of the CIA is significant enough to refuse potentially curative chemotherapy. It is necessary to explore their expectations of the CIA, anticipate reactions to alopecia, and how women intend to prepare for an altered body appearance. Anticipatory coping behavior (ACB) has four levels. These include anticipating, coming to terms with inevitability, becoming ready, and taking control. Studies regarding a woman's critical aspects of ACB towards hair loss and factors influencing it are sparse, especially in India [1]. This study helps identify the factors that influence the ACB towards hair loss. The primary objective of this study was to identify the number of critical aspects of ACB in women undergoing neo-adjuvant CIA for their breast cancer treatment. A secondary objective was to assess the factors associated with anticipatory coping behavior (psychosocial and social impact).

This article was first presented for an undergraduate research award, the Golden Jubilee Short-Term Research Award to undergraduate students (GJSTRAUS), at the Seventh Annual Research Day 2022, Jawaharlal Institute of Postgraduate Medical Education and Research (JIPMER), Pondicherry, India, on 3rd March 2022 (https://jipmer.edu.in/sites/default/files/List%20of%20presentations%207RD%20updated%20.pdf).

## Materials and methods

Study patients

The study included all female breast cancer patients more than 18 years of age admitted for neo-adjuvant chemotherapy. The study excluded patients with recurrent breast carcinoma, those who had already received chemotherapy, with active emotional and cognitive disorders, pre-existing psychiatric illness, those who have recovered from it, not completed even one cycle of chemotherapy, and had prior CIA. Also, the study excluded patients with non-chemotherapy-induced alopecia and alopecia due to other causes like hematological malignancies with generalized metastases, clinical signs of scalp skin metastases, and hereditary alopecia.

Sampling technique

A convenient sampling technique was used. Calculations were done with Open Epi software version 3.0 (Open Source Epidemiologic Statistics for Public Health, www.OpenEpi.com) using the previous study estimate done by Münstedt et al. [1]. It was found that 97% of patients undergoing chemotherapy had alopecia and 73.3% of the patients did not feel as self-confident as before treatment of chemotherapy considering this proportion with 95% confidence interval and 15% relative precision and a dropout rate of 20%, the expected sample size was calculated to be 40 during the two months study period [1].

Study procedure

This prospective observational study was done in the Department of Surgery Jawaharlal Institute of Medical Education and Research (JIPMER), Puducherry, India, from May 2021 to June 2021. Prior approval was taken from the Institutional Ethics Committee (JIP/IEC/2020/028). All patients who fulfilled the inclusion and exclusion criteria were recruited for the study. Written informed consent was obtained from all the patients. Social and demographic data of the patients, stage of carcinoma, type of treatment, and the details of chemotherapy cycles during the initial interview were recorded. ACB before and during each chemotherapy cycle period (minimum one cycle) was assessed using the World Health Organization (WHO) scale.

Study patients received two sets of questionnaires. The first question included baseline demographic data, disease details, and awareness about hair loss. The second question included hair loss pattern and severity of CIA, and the degree of alopecia in a visual analog scale score. The first question is asked before the start of chemotherapy, and the second question is asked at least four weeks after completing the first chemotherapy.

The severity of cancer-induced alopecia is graded by the WHO, grade 0 for none, grade 1 for mild, grade 2 for pronounced, and grade 3 for total alopecia. Furthermore, patients were asked to rate the degree of alopecia in a Visual Analogue Scale (VAS) score ranging from 0 (for no alopecia) to 100 (for total baldness) and details about the ACB pattern. Individual psychosocial and social factors associated with the ACB are documented using a self-administered questionnaire. ACB was categorized into four levels. These include anticipating hair loss, coming to terms with the inevitability of hair loss, becoming ready, and taking control [1]. 

These four themes are correlated with the socio-psychological parameters. The adjustment refers to the psychological processes that occur over time as the individual, and those in their social world, manage, learn from, and adapt to the multitude of changes that have been precipitated by the illness and the treatment, failure of this adjustment leads to deficiency in ACB [2].

## Results

A total of 40 patients were included in the study. All the patients had CIA. Eighteen (45%) patients developed body pain and fatigue, six (15%) patients developed oral ulcers, four (10%) patients developed nausea and vomiting, two (5%) patients developed diarrhea, five (12.5%) patients developed bleeding, and three (7.5%) patients developed some other side effects. Ten (25%) patients were willing to face hair loss. About 14 (35%) patients were afraid to face hair loss. Thirty-two (80%) participants had total hair loss, and 30 (75%) patients had grade 3 hair loss. There was a statistical difference between VAS scores about alopecia before and after chemotherapy (p<0.05) (Table 1).

**Table 1:** Baseline hair loss knowledge among study patients. *Data presented as mean; WHO: World Health Organization; VAS: Visual Analog Scale. Medium (below the shoulder) Good (below the spine of the scapula) Long (below the inferior angle of the scapula)

Parameters	Number (%) (n=40)
Hair growth	
Low (above the shoulder)	3 (7.5)
Medium (below the shoulder)	19 (47.5)
Good (below the spine of the scapula)	14 (35)
Long (below the inferior angle of the scapula)	4 (10)
Side effects of chemotherapy	
Hair loss	40 (100)
	0
Body pain and Fatigue	18 (45)
	22 (55)
Oral ulcers	6 (15)
	34 (85)
Nausea and Vomiting	4 (10)
	36 (90)
Bleeding	5 (12.5)
	35 (87.5)
Diarrhea	2 (5)
	38 (95)
Other	3 (7.5)
	37 (92.5)
Are you willing to face hair loss?	
Yes	10 (25)
No	30 (75)
What is your current mental status of hair loss?	
Afraid	14 (35)
Not Afraid	26 (65)
Hair loss type	
Patchy	10 (25)
Total	30 (75)
WHO grade	
0	0
1	2 (5)
2	8 (20)
3	30 (75)
VAS about alopecia^*^	
At Admission before chemotherapy	20.125
After six weeks of chemotherapy	75.5

Fifteen (37.5%) patients were afraid after noticing hair fall, and 14 (35%) shaved their heads completely. Thirty-four (85%) patients had support from a family member after witnessing the hair fall, which helped them achieve ACB. After the family members, 25 (62.5%) of the patients’ neighbors suggested the patients wear a wig, 13 (32.5%) patients’ friends laughed at them, and two (5%) patients’ relatives ignored the patients after noticing the hair fall. Twenty-three (57.5%) patients tried to cover up their heads; because they felt shame in front of society. Three (7.5%) patients had discussed their hair loss with family members, 14 (35%) patients had discussed their hair loss with friends, and 23 (57.5%) patients did not discuss it with anyone (Table 2).

**Table 2:** Mood affective parameters after hair loss in study patients.

Mood affective parameters	Number (%) (n=40)
Patient's reaction after noticing hair fall	
Loss of confidence	3 (7.50)
Afraid	15 (37.50)
Did not care about hair loss	5 (12.50)
Shaved head completely	14 (35.00)
I did not worry as I knew already.	1 (2.50)
Not affected	2 (5.00)
The first reaction of family members	
Supportive	34 (85.00)
Not supportive	1 (2.50)
No reaction	5 (12.50)
Who noticed first after the patient's family members, and what was their reaction?	
Neighbors suggested wig	25 (62.50)
Friends laughed	13 (32.50)
Relatives avoided	2 (5.00)
Did patients try to cover it up?	
Yes	23 (57.50)
No	17 (42.50)
With whom did patients speak about hair loss?	
Family members	3 (7.50)
Friends	14 (35.00)
Not discussed	23 (57.50)

All the patients (100%) in the study population only thought about hair loss ('anticipating'). About 38 (95%) patients were coming to 'terms with the inevitability of losing hair and were ready to face hair loss; many of the patients took control of hair loss. Thirty-one (78%) patients started to cope with losing hair ('becoming ready'). Almost 37 (93%) patients voluntarily, after noticing hair loss go to the temple they shave their head ('taking control') (Table 3).

**Table 3:** Achievement of anticipatory coping behavior in study patients.

Anticipatory coping behavior	Number (%) (n=40)
If achieved 'anticipating hair loss' reason for that (e.g., oh my god, I am going to lose my hair, I did not think about anything else)	
Yes	40 (100)
No	00
If achieved 'coming to terms with inevitability of hair loss' reason for that (e.g., I did not want chemotherapy. did not want to lose my hair)	
Yes	38 (95)
No	2 (5)
If achieved 'becoming ready' reason for that (e.g., I have already set myself up with one wig, and I have another one in the pipeline coming along)	
Yes	31 (77.5)
No	9 (22.5)
If achieved 'taking control' reason for that (e.g., at what point in treating hair loss might be expected to occur, advice about wearing hats to catch lost hair)	
Yes	13 (32.5)
No	27 (67.5)

Thirty-two (80%) patients felt poor motivation from health care professionals. There were 28 (70%) patients who had a lack of self-confidence, 21 (52.50%) patients who felt shame in front of society, 20 (50%) were insecure about the future, and seven patients who had other social reasons for failure. Nineteen (47.5%) patients were not willing to live with a wig, 37 (92.5%) patients felt less loved by their loved ones, and 38 (95%) patients had poor support from their families (Table 4).

**Table 4:** Reason for failure in study patients due to deficient anticipatory coping behavior.

Reason for failure	Number (%) (n=40)
Poor motivation from healthcare professionals	
Yes	32 (80%)
No	8 (20%)
Lack of self-confidence	
Yes	28 (70%)
No	12 (30%)
Feeling shame in front of society.	
Yes	21 (52.50%)
No	19 (47.50%)
Insecure thoughts of future	
Yes	20 (50%)
No	20 (50%)
Other social factors	
Yes	7 (17.50%)
No	33 (82.50%)
Not willing to live with the wig	
Yes	19 (47.50%)
No	21 (52.50%)
Feeling different from loved ones.	
Yes	37 (92.50%)
No	3 (7.25%)
Poor support from family	
Yes	38 (95.00)
No	2 (5.00%)

## Discussion

Despite numerous unpleasant side effects of chemotherapy, such as vomiting, nausea, and tiredness, a previous study found that hair loss was the most traumatic aspect of the treatment for 46% of respondents [3]. Nursing understands possible illness-related changes in self-concept and how changes affect the healing process across the illness/wellness continuum [4]. These activities include resource accumulation (information), initial appraisal (assessment of the impact of the event), initial coping efforts (actions to prevent or minimize the event), and elicitation and use of feedback [5,6].

Out of the total, 52.5% of the patients felt shame in front of society; that is the reason for deficient ACB. 34.5% of participants were afraid to face hair loss. The remaining patients were supported by family members, which helped them to achieve anticipatory coping behavior. This may be due to a lower level of education and poor socio-economic background. Most of the patients felt that proper instruction from the clinicians and family support would help achieve ACB. 

The most common reaction of patients noticing hair loss was being afraid (37.5%) and losing confidence. Of those who saw hair loss, 35% shaved their heads. Only a few were not worried that they were losing hair, probably due to the achievement of the ACB. Most of the study patients' family members were supportive, which helped them achieve anticipatory coping behavior. Some of the patients spoke to family members or friends, but a few others were not open to discussing hair loss. Family members give moral support to the patient in their difficult times. The neighbors of the patients suggested wearing a wig.

Some of the friends of the patients had laughed at them for alopecia. Some of the relatives of the patients avoided talking to the patients. These kinds of behavior from relatives and friends may build a social stigma about cancer patients undergoing chemotherapy. Patients who are facing all these difficulties from society feel dejected. Moreover, many patients tried to cover up their heads because they felt shame in front of the community. Patients, along with friends and relatives, should be counseled and encouraged to discuss hair loss with their relatives or friends, and they should be advised not to worry about what society thinks and to be afraid of losing hair [7].

None of the patients in the study population solely thought about hair loss. A few of them were coming to terms with the inevitability of losing hair and were becoming ready to face hair loss. Many of the patients achieved taking control of hair loss. Some patients coped by voluntarily shaving their heads at the temple, while others remained worried. A previous study observed CIA severely affects the patient's quality of life [8]. Another study demonstrated that patients need information and support for chemotherapy-related problems [9]. Physicians can advise such patients that losing hair is not a detrimental health problem and encourage them to use hats.

The study patients' main reasons for lack of ACB were shame in society, insecure thoughts about the future, and unwillingness to live with a wig. Patients should be counseled on managing the social implications of the CIA, and other avenues of support may be suggested to those who feel estranged from their families. Since some patients feel poor motivation from health care professionals is also a reason, it must be corrected so that in the future for other patients, we can encourage them and help them to achieve coping behavior.

The limitations of this study are that it is a prospective analytic study with a small sample size of the population and a failure to analyze other socioeconomic factors. Larger sample sizes with an analysis of patient ACB in a different stage of hair loss will give more consolidated results. Further multi-institutional prospective studies are required to assess the poor motivation of health care professionals to discover what changes are needed to achieve the target ACB. Further studies can be conducted using a computerized program to endorse patients with education and information for the CIA [10]. Few studies have also demonstrated that scalp cooling may reduce the CIA, which can be advised to patients [11].

## Conclusions

The main reasons for the lack of ACB are the lack of support from treating clinicians and family members. Once patients got support from family members, they achieved “taking control” of hair loss in a better way. Few patients started to cope with losing hair by shaving their heads with a temple or wig, or hat. While others just worried about hair loss and did not find ways to cope with the hair loss. The clinician can encourage them adequately so that patients will get self-confidence, secure thoughts about the future, and live with wigs or hats for time being, as the CIA is temporary.
